# A new precision medicine initiative at the dawn of exascale computing

**DOI:** 10.1038/s41392-020-00420-3

**Published:** 2021-01-06

**Authors:** Ruth Nussinov, Hyunbum Jang, Guy Nir, Chung-Jung Tsai, Feixiong Cheng

**Affiliations:** 1 Computational Structural Biology Section, Frederick National Laboratory for Cancer Research in the Laboratory of Cancer Immunometabolism, National Cancer Institute, Frederick, MD 21702 USA; 2 Department of Human Molecular Genetics and Biochemistry, Sackler School of Medicine, Tel Aviv University, Tel Aviv, 69978 Israel; 3 Department of Genetics, Harvard Medical School, Boston, MA 02115 USA; 4 Wyss Institute for Biologically Inspired Engineering, Harvard University, Boston, MA 02115 USA; 5 Genomic Medicine Institute, Lerner Research Institute, Cleveland Clinic, Cleveland, OH 44106 USA; 6 Department of Molecular Medicine, Cleveland Clinic Lerner College of Medicine, Case Western Reserve University, Cleveland, OH 44195 USA; 7 Present Address: Department of Biochemistry & Molecular Biology, Department of Neuroscience, Cell Biology and Anatomy, Sealy Center for Structural Biology and Molecular Biophysics, University of Texas Medical Branch, Galveston, TX 77555 USA

**Keywords:** Predictive medicine, Biophysics, Cancer

## Abstract

Which signaling pathway and protein to select to mitigate the patient’s expected drug resistance? The number of possibilities facing the physician is massive, and the drug combination should fit the patient status. Here, we briefly review current approaches and data and map an innovative patient-specific strategy to forecast drug resistance targets that centers on parallel (or redundant) proliferation pathways in specialized cells. It considers the availability of each protein in each pathway in the specific cell, its activating mutations, and the chromatin accessibility of its encoding gene. The construction of the resulting Proliferation Pathway Network Atlas will harness the emerging exascale computing and advanced artificial intelligence (AI) methods for therapeutic development. Merging the resulting set of targets, pathways, and proteins, with current strategies will augment the choice for the attending physicians to thwart resistance.

## Introduction

**Fig. 1:**
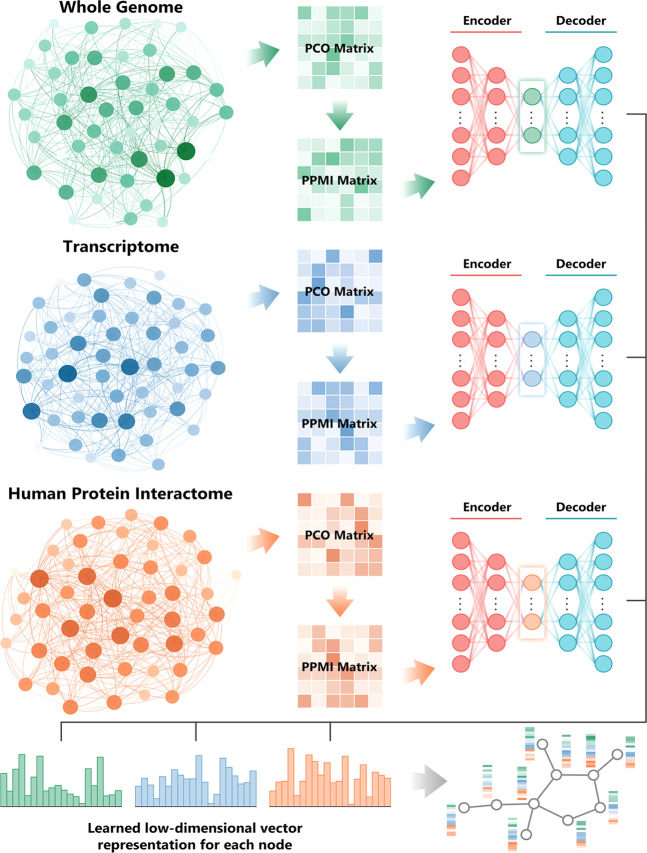
A workflow illustrating an artificial intelligence-based, exascale computing framework for integrative analysis of multi-omics data for precision cancer medicine. PCO, probabilistic co-occurrence; PPMI, positive pointwise mutual information. The detailed description about a deep neural network-based multi-omics and network integration can be found in a recent study^81^

Precision medicine aims to identify patient-specific drug targets.^1–5^ To date, the approaches have largely focused on (i) identification of proteins with driver mutations; the mutations can be strong drivers, weak drivers, rare drivers, or latent drivers;^2,5–11^ (ii) decision on how to target: should the mutant protein be targeted with a combination of drugs, e.g. one orthosteric and the other allosteric, or should a second protein also be targeted, in which case should the second protein be from the same pathway (the more frequent case) or from a different pathway.^12^ If from a different (redundant, or parallel) pathway, the protein is generally selected based on the physician’s prior knowledge; and finally, once identified, (iii) selection of drugs targeting these. Drug discovery can be via large-scale screening, in silico docking, structure-based drug design, or drug repurposing.^13–18^ The approaches rely on vast quantities of data, high-resolution structural data, highly efficient state-of-the-art algorithms to sift through these and large-scale scientific computation.^1^ They are mapped in the diagram in Fig. 1. These approaches empowered significant progress since the launch of the precision medicine initiative, with breakthrough discoveries identifying activating mutations in key oncogenic proteins and their isoforms, their patterns and mechanisms.^19^ However, the complexity and challenge of identifying drug resistance targets call for broadening the current strategies and marshalling new ones from a different standpoint.

The framework outlined here maps a conceptually innovative complementary Precision Medicine Initiative that embraces the principles of cell proliferation to counter drug resistance. Different than current strategies it proposes (i) to identify or predict all potential *proliferation pathways* in the cell. Proliferation pathways constitute the ‘stockpile’ of drug resistance pathways; (ii) to investigate the chromatin accessibility of genes encoding each protein in each proliferation pathway in the specific cell to confirm pathway availability in drug resistance, as well as their cell-specific expression levels; (iii) to identify their driver mutations, and the mechanisms of pathway activation; and finally, exploiting artificial intelligence (AI) methodologies, to (iv) integrate multi-omics cancer data and networks’ perturbations for therapeutic development. Thus, it aims to stop cell proliferation by identifying all possible proliferation pathways and *predicting which gene is likely to become the next driver* of cancer in the specific patient cell. This innovative and comprehensive strategy is computationally intensive and proposes to exploit the emerging exascale computing in the last two steps. We expect that a Proliferation Pathway Atlas incorporating such data would be an invaluable resource to the community.

## Proliferation pathways are critical in cancer

Proliferation pathways link the cellular environment to the cell cycle. Genetic (mutations) and epigenetic alterations can lead to pathway hyperactivation, fueling cancer progression, as does inactivation of tumor suppressors. Protein-protein interactions of major cancer drivers are enriched in mutations, hijacking pro-proliferative signaling networks.

Pathways crosstalk. Crosstalk emerges due to shared interactions and elements.^20,21^ It can influence their expression level and function. Under physiological conditions, crosstalk enables cells to cope with perturbations of homeostasis. In drug resistance, inhibition of a signaling pathway can promote activation of a survival pathway that bypasses the inhibited pathway. Insight into connections between signaling pathways and foresight into their distinct activation can be powerful in the treatment of cancer.^22^

### What distinguishes proliferation pathways from other signaling pathways?

The Ras/phosphoinositide 3-kinase (PI3K)/Akt and Ras/extracellular signal-regulated kinase (ERK) pathways provide good examples of proliferation pathways.^23,24^ Ras is activated by stimulated receptor tyrosine kinases (RTKs). Ras mutants are involved in roughly a third of the cancers. The identity of the other two-third proliferation pathways is only partially known.

**Fig. 2:**
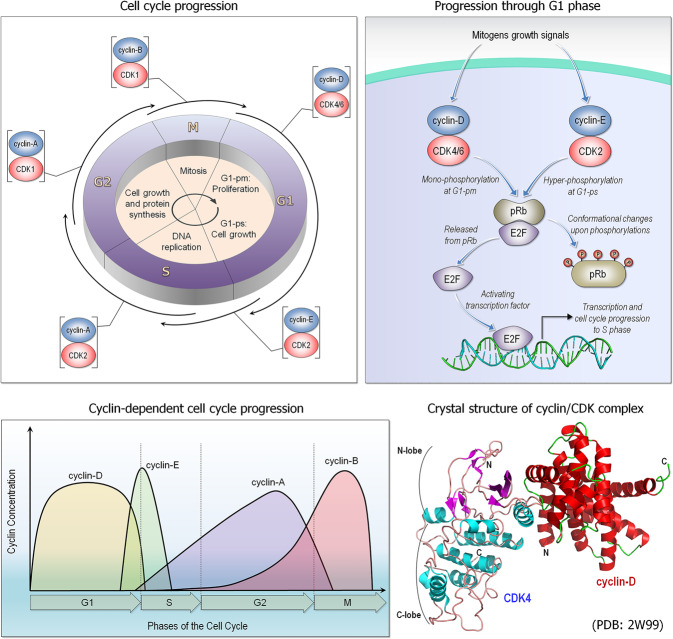
Cyclin-dependent kinases (CDKs) cell cycle. CDKs and their cyclin partners form complexes, regulating the progression through the cell cycle (upper left panel). In early G1 phase (G1-pm), retinoblastoma protein (pRb) becomes phosphorylated by the complex, a pair of cyclin-D and CDK4/6 (upper right panel). In late G1 phase (G1-ps), pRb is hyper-phosphorylated by cyclin-E/CDK2 complex, undergoing a large conformational change. This conformational change fails to assemble with E2F, a transcription factor, which promotes to progress the G1/S transition. Cyclins bind with the dependent kinases and their concentration varies during the cell cycle (lower left panel). A crystal structure illustrates an example of cyclin/CDK complex (PDB: 2W99) (lower right panel). Apo-CDKs exhibit little kinase activity; CDKs become active kinases when interacting with the regulatory protein called a cyclin

Proliferation involves cell growth and division. Proliferation can take place through many pathways and is particularly active during development. It is also essential in adult homeostasis. Signaling pathways that control cell proliferation^25^ can act by linking the cellular environment to progression through the G1 (Gap 1) phase of the cell cycle (Fig. 2). Progression through G1 is controlled by retinoblastoma protein (pRb) whose phosphorylation by the G1 cyclin-dependent kinases (CDKs) promote passage of the cell cycle to the S (Synthesis) phase. The pRb pathway (thus G1) is mainly regulated by cyclins and CDK inhibitors with inputs from major cellular signaling pathways. pRb tumor suppressor binds to the E2F1 transcription factor (TF), repressing the G1/S transition; phosphorylation of pRb proteins by CDKs liberates E2F, promoting the transition to S phase.

### Criteria for identifying proliferation pathways

A pathway that promotes cell proliferation (i) can lead to activation of TFs that induce expression of proteins acting in multiple pathways, including oncogenic functions such as proliferation and survival, with some of these (ii) entering the cell cycle. Cyclin-D, whose synthesis is initiated during the cell cycle G1 phase and is involved in regulating cell cycle progression provides an example (Fig. 2). The cyclin-D/CDK4 complex, which consists of cyclin-D and CDK4, or CDK6, a serine-threonine kinase, is essential for the progression of the cell from the G1 to the S phase, for the Start or G1/S checkpoint. Some proteins control operations critical for cell cycle progression. Cyclin-D transcription is activated through the growth factor-stimulated RTK proliferation pathway which expresses Myc, a TF that controls transcription of several cell cycle-regulating genes, including cyclin-D. Myc promotes the cell cycle primarily through its role in cellular growth control. c-Myc target genes include regulators of cell growth; but also, those functioning in cell division pathways. Among c-Myc target genes that regulate cell growth are those associated with ribosomal protein transcription and translation, including translation initiation factors such as eukaryotic translation initiation factor 4E (eIF4E). Active RTK signals through the two major signaling pathways; c-Myc is involved in both. Notably, a proliferation pathway, such as MAPK can also activate gene sets for immune response.^26^

## Examples of proliferation pathways

Wnt/β-catenin, Notch, Hedgehog, transforming growth factor β (TGF-β) and Hippo are implicated in developmental processes and proliferation. Janus kinase/signal transducer and activator of transcription (JAK/STAT) is an example of a proliferation pathway through a cytokine receptor (IL7). Here we focus on development-related pathways and discuss the first three. Embryogenesis and tumorigenesis share coordinated mechanisms of proliferation, differentiation, and migration.^27^

### Wnt/β-catenin signaling

**Fig. 3:**
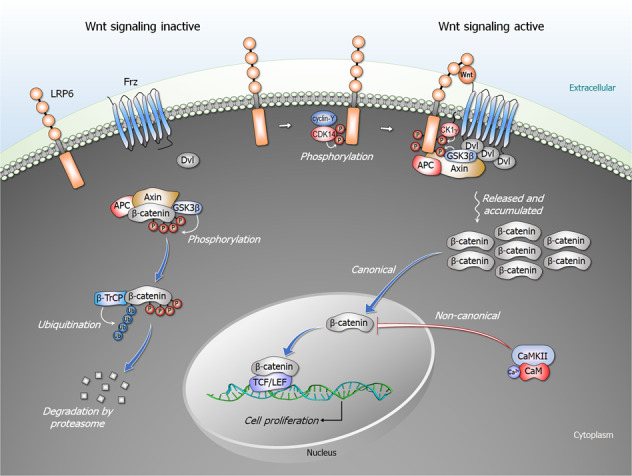
The Wnt signaling pathways. In the absence of Wnt signal, the destruction complex constituted by Axin, adenomatosis polyposis coli (APC), and glycogen synthase kinase 3β (GSK3β) leads to phosphorylation of β-catenin by GSK3β in the cytoplasm. The phosphorylated β-catenin is subsequently ubiquitinated by β-TrCP and targeted for proteasomal degradation. The canonical Wnt/β-catenin pathway is activated by upon binding of Wnt to its Frizzled (Frz) receptor and low-density lipoprotein receptor-related protein 6 (LRP6) co-receptor. LRP6 phosphorylated by cyclin-Y/CDK14 complex, GSK3β, and casein kinase 1γ (CK1γ) recruits the destruction complex and Dishevelled (Dsh, Dvl in mammals) to the plasma membrane. Dvl is activated through polymerization, inhibiting the destruction complex. This results in accumulation of unphosphorylated β-catenin in the cytoplasm and its subsequent translocation into the nucleus, leading to cell proliferation. In the non-canonical Wnt/calcium pathway, calmodulin-bound CaMKII (calcium/calmodulin-dependent protein kinase II) negatively regulates the canonical β-catenin/TCF/LEF signaling through phosphorylation of TCF, which inhibits β-catenin/TCF-mediated transcription

The Wnt signaling cascade is a main regulator of development, controlling the growth of embryonic stem cells and adult cell specialization (Fig. 3). The pathway is also frequently active in cancer.^28^ Wnt growth factors alter gene expression by stimulating different classes of receptors. They lead to cell proliferation through their impact on the cell cycle.^29^ Wnt pathway components, such as β-catenin, Dishevelled (Dsh, Dvl in mammals), Frizzled (Frz, a Wnt receptor), low-density lipoprotein receptor-related protein 6 (LRP6, a Wnt co-receptor), and Axin have been associated with cell cycle regulation, centrosome biology, and cell division. Several Wnt pathway components play essential roles during mitosis, which is proposed to also regulate Wnt signaling via cyclin-Y/CDK14 phosphorylation of LRP6.^30^ They also control cell morphogenesis, affecting the cytoskeleton and the mitotic spindle. Wnt-stimulated signaling activates β-catenin which interacts with DNA-bound TFs of the T-cell factor (TCF) family. β-catenin switches inactive TCF into a transcriptional activator of its target genes.^31^ Chromatin remodeling complexes can bind β-catenin and promote transcriptional activation of TCF-responsive reporter genes. Transcriptional co-activators, such as p300 and cAMP-response element binding protein (CREB) can alter chromatin structure through histone acetyltransferase to stimulate transcriptional activity. In the absence of a Wnt signal, β-catenin is degraded by a complex which includes the Axin scaffold protein, glycogen synthase kinase 3β (GSK3β), and adenomatous polyposis coli (APC). TCF is bound to the Groucho repressor; binding of Wnt to its receptors induces dissociation of the complex. β-catenin binds TCF in the nucleus.

### Hedgehog signaling

Hedgehog communicates between cells. It is important for organ development, regeneration and homeostasis; it is frequently modulated in cancer.^27^ It cross-talks with e.g. transforming growth factor β (TGFβs), Wnt, Notch, and the Sonic hedgehog (Shh). The Shh pathway can involve canonical or non-canonical signaling. The first is receptor ligand-dependent when Shh binds to Ptch (a 12-transmembrane protein) at the membrane; the second is through downstream smoothened (Smo).^32^ Smo regulates Gli transcription factors processing and activation, which activate target genes. Non-canonical activation is Gli-independent. Hedgehog signaling upregulates multiple proteins, including N-Myc (a member of the Myc family), forkhead box M1 (FoxM1), and Cdc25B, which activates the cyclin-dependent kinase *CDC2*. It also upregulates *CCND1*, *CCND2*, and *CCNE*. Cyclin-D1, cyclin-D2, and cyclin-E which drive cell-cycle progression at the G1/S phase, while FoxM1, cyclin-B1, and Cdc25B act at the G2/M (mitotic) phase. Thus, hedgehog signals drive cell-cycle progression through multiple cell cycle regulators.

### Notch signaling

Notch signaling takes place via cell-cell communication, where transmembrane ligands on one cell activate those of the other. The cleaved receptor is translocated to the nucleus.^33^ Notch intracellular domain (NICD) forms a trimeric complex with CSL (CBF1, Suppressor of Hairless, Lag-1; a transcription factor that activates genes downstream in the Notch pathway) and Mastermind-like (MAML) transcriptional coactivator, which converts CSL from a repressor to an activator and initiates transcription of Notch downstream target genes. In the absence of Notch signaling, CSL represses transcription; following activation by Notch, it is converted into a transcriptional activator and activates transcription of the same genes. Notch signaling with its CSL cofactor can maintain cells in an undifferentiated state, consequently associated with cancer. It controls cell lineage and tissue development, blocking differentiation thus retaining stem or progenitor cells, or governing the balance between cell fates. Notch signaling mediates G1/S cell-cycle progression in T-cells via cyclin-D3 and its dependent kinases and activates cell cycle reentry and progression in quiescent cardiomyocytes. Notch signaling acts before cell division to promote asymmetric cleavage and cell fate of neural precursor cells; its activation can inhibit proliferation of endothelial cells by delaying cyclin-D/CDK4-mediated phosphorylation of the retinoblastoma protein. It also regulates variant cell cycles to control cell size^34^ and more.

### EGFR signaling

Epidermal growth factor receptor (EGFR) pathway is a classic example of proliferation pathway that can lead to G1 cell cycle progression, through cyclin-D expression, CDK4/6 activation, and the repression of cyclin-dependent kinase inhibitor proteins (CDKi) by EGFR signaling pathways.

### Selection of the proliferation pathway to drug

Halting proliferation by drugging the pathway most likely to become the next driver in the patient cell is a powerful and compelling amplification of current therapeutic approaches. It considers cancer evolution dynamics which to date has been missing. The challenge is however in the knowledge of (i) all possible proliferation pathways, (ii) the accessibility of each gene encoding a protein in each pathway in the specific cancer cell, including (iii) expression data, and (iv) the driver mutations in each gene.

## Genes of targeted pathways should be accessible

To be a good drug candidate, the proteins in the proliferation pathway should be available in the specific cell. This requires that the genes encoding the pathway proteins are accessible to the transcription machinery or can become accessible upon a ‘modest’ change in the chromatin structure. Not all proteins are expressed in all cells. Chromatin availability status is cell type, lineage and state-dependent.^12,35^ Genes active in developmental or embryonic pathways can become densely packed in the chromatin and inaccessible. Further, because signaling in a skin cell differs from that in a kidney cell, proliferation pathways in drug resistance are likely to differ between these cells.^36^ Oncogenic cells manifest tissue-specific tendencies,^36,37^ with distinct cells having preferred proliferation profiles. Accessibility is controlled by cell-specific chromatin-binding factors,^38,39^ including e.g. pioneer transcription factors that locally unfold the condensed chromatin and nucleosomes. Accessibility can also be regulated by the proliferation pathway itself, as in the case of Notch^33^ and its epigenetics.^40^

Experimental accessibility data are limited. Predicting the three-dimensional genome organization and chromatin accessibility is also challenging. High-resolution structural data provide structural detail, allow mapping of genomes, insight into effects of mutations and dysregulation that traditional methods that identify the genes with active histone modification markers, such as H3K27ac, H3K4ac3 are unable to provide. Simulations with parameterization based on the free-energy landscape theory,^41,42^ genomics and epigenomics data, reproduced chromosome conformation capture data (Hi-C)^12,43–47^ and super-resolution microscopy.^42,48,49^ They permitted predicting chromatin structures at 5 kilobase resolution starting from genomics and epigenomics data that are available for hundreds of cell types, including cancer cells.^42^ Integration of Hi-C data with conventional microscopy led to more accurate prediction of genome organization.^50^ More recently, Hi-C data and super-resolution imaging were brought together through integrative modeling of genomic regions (IMGR), thus achieving high spatial and genomic resolution, while maintaining the single-cell identity.^51–53^ IMGR can be broadly divided into three steps. In step one, models are constructed of Hi-C data.^54,55^ In step two, these models are rigidly fitted onto structures resolved by super-resolution microscopy. The top 5% that fit the most qualify to the next step, which is the flexible fitting. In flexible fitting, the polymer chains are allowed to swivel around TAD borders, which are expected to be more flexible. The model that best fits each super-resolved structure is chosen. Such a technology promotes optimism that a precision level that unearths the chromatin status of driver genes is reachable; *genes with sparse chromatin density would suggest that they are drug resistance candidates*. Integrative successes promise increasingly detailed mapping of dynamic chromatin maps of single cells.

**Fig. 4:**
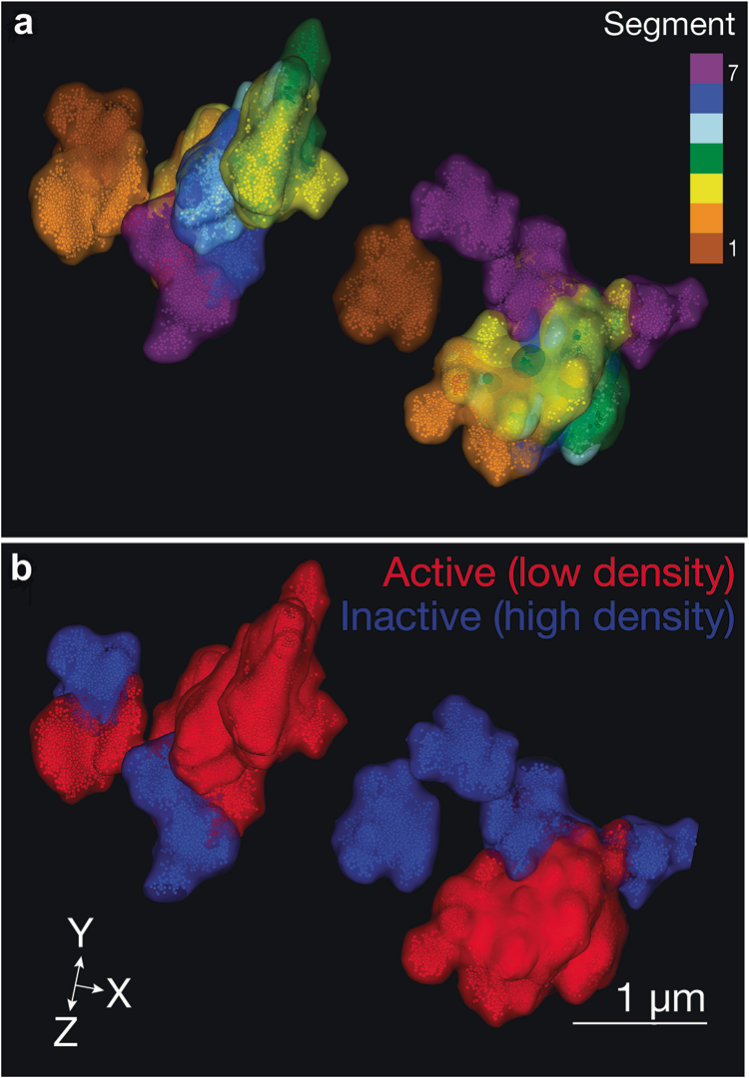
OligoSTORM image of chromatin density. (a) Seven chromosomal segments (chr19:8.68–15.2; hg19) rendered as isosuurface clusters in a PGP1 fibroblast. Each color represents a different chromosomal segment. (b) The seven segments color-coded according to their transcriptional activity and epigenetics marks^51^ (blue—inactive, red—active). Here, the density of the inactive is higher than the active chromatin

IMGR is especially beneficial when integrating with images (Fig. 4). Here, we focus on 7 chromosomal segments out of the 9 imaged using sequential OligoSTORM, and color-code them as either active (red) or inactive (blue).^51^ OligoSTORM^52,56^ is the integration of Oligopaints Fluorescence in situ Hybridization (FISH) probes,^57^ with the super-resolution technology called Stochastic Optical Reconstruction Microscopy (STORM).^58^ Sequential OligoSTORM^51,53,59^ allows imaging of multiple genomic loci, going much beyond the limitations of spectral resolution (Fig. 4A). Even though these chromosomal segments were imaged at the ~ Mb scale, with IMGR, their genomic resolution can improve to 10 kb and better, which is two orders of magnitude higher for some of these segments. Interestingly, the density of the inactive chromatin in this PGP1f (Personal Genome Project, participant 1 fibroblasts) nucleus is higher than that of the active chromatin (Fig. 4B). Drugs may find active, cell-type specific, chromatin target more efficiently. OligoSTORM gene-specific visualizing technologies, or IMGR, can learn whether gene accessibility is influential in successful drug therapy. The efficiency of drug therapy might also be dependent upon the structural variation between homologous chromosomes.^51^

## Identifying driver mutations with exascale computing

**Fig. 5:**
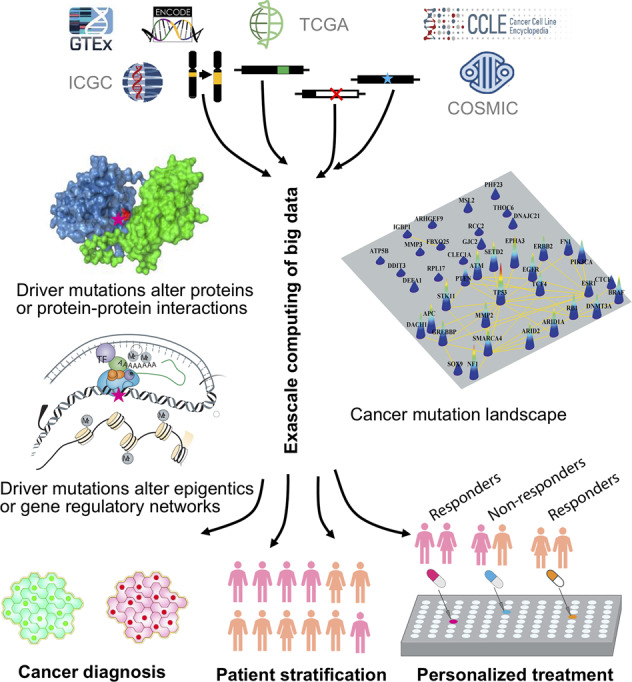
A proposed atlas and its associated portal for precision cancer medicine using exascale computing of “big” data

Proliferation pathways are activated by driver mutations. Their identification involves algorithmic strategies, statistical evaluation and databases.^2,19,60–64^ Since the methods are statistics-based, the mutations are mostly identified based on their frequencies of occurrence. Recently, however, an increasing number of statistically rare mutations were identified in patients, raising the question of how to identify rare, and weak drivers which are often observed only in certain tissues thus overall infrequent.^5,19,62,65–72^ K-Ras4B^A146T^ is one example where the mechanism is understood. Different than K-Ras4B^G12D^, a strong driver that blocks GTP hydrolysis and is expressed in many cancers, including pancreatic and colon, the weaker K-Ras4B^A146T^ which acts by promoting guanine nucleotide exchange factor (GEF)-mediated GDP by GTP exchange, transforms colon but is not sufficiently powerful in transforming pancreatic cancer cells.^73^ “Latent” mutations, that need an emerging ‘helper’ mutation with additive effects for observable pathological consequences are especially challenging to identify. Mechanistically, whether frequent or rare, mutations that release autoinhibition are often driver mutations;^63^ clusters of mutations also tend to contain drivers, including rare, and latent.^5,19,62^ Identification of driver mutations, including weak, rare and latent, in each protein in all proliferation pathways requires immense computational power. These mutations are determined not based on their statistics, but by their ability to shift the protein conformation from an inactive to the active state. Identification of each mutation in each protein necessitates powerful computing to observe whether it executes this shift, expressed by conformational change. Such computing power is forecast to reach to scientific community. Exascale computing systems are capable of a billion (i.e. a quintillion) calculations per second. This scale permits executing such long timescales explicit solvent simulations which are required to capture the redistributions of the ensembles. These indicate the population time of conformations where the mutation switches the protein from the inactive to the active state. Figure 5 illustrates why massive compute time is necessary.

## Artificial intelligence, multi-omics data, and network perturbations for therapeutic development

The human genome project accelerated genetic and genomic studies such as The Cancer Genome Atlas (TCGA) to inform precision medicine drug discovery.^1^ The underlying hypothesis of cancer systems biology is that sub-cellular networks gradually rewire throughout disease initiation, progression, and maintenance, leading to progressive shifts of local and global network properties and systems states,^74^ including protein-protein interactions and gene regulatory network, all controlling cancer initiation and drug responses (Fig. 5). Genome alterations, amplification, deletion, translocation, and mutations can only be selected for in cells if they encode changes, or perturbations, in the human interactome and systems properties of the affected cells.^75,76^ Personalized treatment needs to be designed to deal with such perturbations; rather than only with genomic events. Analysis of over 2.5 million nonsynonymous somatic mutations derived from 6,789 tumor exomes across 14 cancer types from TCGA, showed that Individualized Network-based Co-Mutation (INCM)-inferred putative genetic interactions are correlated with patient survival and drug responses in cancer cell lines.^75^ Drug-target network analysis revealed candidate therapeutic pathways that target tumor vulnerabilities and identified several potential pharmacogenomics biomarkers. A Genome-wide Positioning Systems network (GPSnet) algorithm incorporated individual patient’s DNA and RNA profiles into the human protein-protein interactome network to prioritize targets and repurposed drugs for cancer.^14^ A GPSnet-predicted and experimentally validated drug, ouabain, revealed potential antitumor activities in lung adenocarcinoma by uniquely targeting a HIF1α/LEO1-mediated cell metabolism pathway.^14^

The human interactome networks already contributed to understanding tumorigenesis and rapid identification of driver genes in human cancer and drug treatment.^1,15,77,78^ Cancer networks, and broadly sub-cellular systems, require information and models at multi-dimensional levels, including cells, tissues, organs, and organisms, which are missing in traditional computational approaches. Cancer therapy is moving from drug-centered to patient-centered approach. This requires paradigm shifts along the entire drug development process and multi-omics data integration. The increase in data (including DNA/RNA sequencing data) and the difficulty of data analysis, will also be aided by exascale computing. Advances in AI have been applied to cancer medicine, particularly in large-scale, integrative analyses of multi-omics and biological networks. Still, development and application of AI methods in precision medicine are still in its infancy.

Cancer data come from high-dimensional sources, electronic health care records (imaging, laboratory results, diagnosis codes), genetic testing, among others. An oncologist has to evaluate vast amounts of information, including the patient’s history, family history, genomic sequences, medications, and more, to guide rapid clinical decision. Among the multiple AI techniques, deep neural networks have gained attention in precision cancer medicine, especially for imaging data analysis^79,80^ and complex biological network integration.^16,81^ Saltz et al. presented convolutional neural network (CNN) models to analyze 5,200 digital images from 13 cancer types.^79^ They demonstrated that tumor-infiltrating lymphocyte maps identified by CNN models were correlated with patient survival, tumor types, and immune profiles.^79^ A one-class logistic regression (OCLR) machine-learning algorithm incorporated transcriptomic and epigenetic profiles from cancer patients for assessing the degree of oncogenic dedifferentiation.^82^ OCLR identified previously undiscovered biological mechanisms associated with the dedifferentiated oncogenic state quantified by stemness indices, a key measurement of cancer progression.^82^ Indices predicted by OCLR revealed novel targets and possible targeted therapies by specifically targeting tumor differentiation.^82^

AI approaches excel at automatically recognizing complex patterns in multi-omics data and providing quantitative assessment of genetic regions, omic layers, and pathways associated with tumorigenesis and precision medicine drug discovery (Fig. 1). deepDTnet, a network-based deep learning methodology was developed for novel target identification and drug repurposing via a heterogeneous drug-gene-disease network embedding 15 types of chemical, genomic, phenotypic, and cellular network profiles.^81^ DCell, a visible neural network embedded in the hierarchical structure of 2526 subsystems comprising a eukaryotic cell,^83^ showed consistent results with laboratory observations when evaluated on several million genotypes.^83^ Its framework may be applied to tumor cells although they are highly complex systems with millions of components and interactions. An AI-based, exascale computing framework that incorporates genome/transcriptome/proteome data, human protein-protein interactome, public drug-target databases (Fig. 1), along with functional validation or patient data validation offers powerful tools for accelerating precision cancer medicine.

Coupled with identification of mutations, enabled by powerful exascale computing at the single protein level, can create a comprehensive and rounded computational framework, whose organization will integrate all components.

## Conclusions: stop cell proliferation

The potential of precision medicine to sustain human health has captivated the imagination of the scientists and the public. The National Cancer Institute described precision medicine as “an approach to patient care that allows doctors to select treatments that are most likely to help patients based on a genetic understanding of their disease”. However, *exactly how to select* has been unclear. The number of possibilities is massive, and the drug combination should fit the patient status. Significant progress has been made since the launch of the precision medicine initiative. However, to date its success has been limited. A major reason is the emergence of drug resistance.

*Here we map a new concept: stopping cancer cell proliferation by targeting the proliferation pathway and genes that are likely to be the next drivers in the expected emergence of drug resistance*. Current technologies, which can already obtain gene-scale resolution of chromatin increasingly allow forecasting such set of drug resistance targets through identification of proliferation pathways and the accessible genes encoding them. While here we focus on proliferation pathways, for completeness, in the future survival pathways and others critical in drug resistance should also be included.

In the biological sciences, exascale computing in the next decade is expected to be dominated by hybrid modeling, molecular dynamics, free-energy simulations, drug design, and discovery, and modeling the behavior of molecular assemblies and cell actions exploiting imaging at different scales. The concept described here fits well into these capabilities aiming to arrest cell proliferation in drug resistance.
